# On the Performance of a Ready-to-Use Electrospun Sulfonated Poly(Ether Ether Ketone) Membrane Adsorber

**DOI:** 10.3390/membranes13060543

**Published:** 2023-05-23

**Authors:** Niki Joosten, Weronika Wyrębak, Albert Schenning, Kitty Nijmeijer, Zandrie Borneman

**Affiliations:** 1 Membrane Materials and Processes, Department of Chemical Engineering and Chemistry, Eindhoven University of Technology, 5600 MB Eindhoven, The Netherlandsd.c.nijmeijer@tue.nl (K.N.); 2 Stimuli-responsive Functional Materials and Devices, Department of Chemical Engineering and Chemistry, Eindhoven University of Technology, 5600 MB Eindhoven, The Netherlands; 3 Wetsus, European Centre of Excellence for Sustainable Water Technology, Oostergoweg 9, 8911 MA Leeuwarden, The Netherlands

**Keywords:** membrane adsorber, sulfonated poly(ether ether ketone) (sPEEK), electrospinning, lysozyme, dynamic adsorption capacity, fiber diameter, functional-group density, sulfonation degree, electrostatic interactions, specific surface area

## Abstract

Motivated by the need for efficient purification methods for the recovery of valuable resources, we developed a wire-electrospun membrane adsorber without the need for post-modification. The relationship between the fiber structure, functional-group density, and performance of electrospun sulfonated poly(ether ether ketone) (sPEEK) membrane adsorbers was explored. The sulfonate groups enable selective binding of lysozyme at neutral pH through electrostatic interactions. Our results show a dynamic lysozyme adsorption capacity of 59.3 mg/g at 10% breakthrough, which is independent of the flow velocity confirming dominant convective mass transport. Membrane adsorbers with three different fiber diameters (measured by SEM) were fabricated by altering the concentration of the polymer solution. The specific surface area as measured with BET and the dynamic adsorption capacity were minimally affected by variations in fiber diameter, offering membrane adsorbers with consistent performance. To study the effect of functional-group density, membrane adsorbers from sPEEK with different sulfonation degrees (52%, 62%, and 72%) were fabricated. Despite the increased functional-group density, the dynamic adsorption capacity did not increase accordingly. However, in all presented cases, at least a monolayer coverage was obtained, demonstrating ample functional groups available within the area occupied by a lysozyme molecule. Our study showcases a ready-to-use membrane adsorber for the recovery of positively charged molecules, using lysozyme as a model protein, with potential applications in removing heavy metals, dyes, and pharmaceutical components from process streams. Furthermore, this study highlights factors, such as fiber diameter and functional-group density, for optimizing the membrane adsorber’s performance.

## 1. Introduction

Our resource consumption surpasses the earth’s replenishment rate. To prevent depletion and ensure long-term sustainability, a transition from a linear to a circular economy is necessary [1]. Looking especially at the key element of water, a circular economy requires the purification of water to safeguard clean drinking water and the recovery of the valuable resources it contains [2]. Currently, many valuable resources are lost in discarded rest streams due to a lack of cost-effective recovery technologies [3]. In many cases, the concentration of the valuable resource is too low, resulting in high energy costs, or the rest streams are contaminated, increasing the purification process costs [3]. Additionally, every industry has unique resources in its rest stream, such as proteins in the dairy industry, ionic species (e.g., nitrogen, phosphorous, and potassium) in the agricultural industry, and dyes in the textile industry [4,5]. These factors make it challenging to find a single, efficient technology to recover and valorize these resources [6,7]. Therefore, an efficient, and especially versatile, purification technology is needed that can be tailored to the needs of various industries.

Traditionally, adsorption processes using packed-bed column technology are frequently applied to remove target components or impurities from aqueous streams [8]. However, significant limitations involving high pressure drop and low throughput separation (the amount of material processed per unit time), seriously impede the development and scale-up of this technology [8,9]. In addition, coarse impurities can easily clog the column, making this technology unsuitable for streams with high-mass components [10]. To overcome these limitations, several developments in column technology have been made.

One such development is reducing the particle size in the packed bed, which increases the adsorption capacity. By reducing the size of the porous particle, the diffusion length is shortened, making the adsorption sites more accessible and increasing the throughput [11]. However, this also intensifies the limitations of clogging and high pressure drop, leading to column deformation and channeling. This results in an early breakthrough, which implies noncomplete utilization of the adsorption capacity [12].

Nonporous and/or core-shell rigid particles, on the other hand, offer the advantage of greater robustness and lower pressure drop [13]. Solute diffusion is no longer a limiting factor due to the absence of pores [8]. Unfortunately, these particles have a lower surface area, which results in a lower capacity [13].

Perfusive or super-porous particles have been developed to increase convective mass transport and reduce the diffusive dependency of the purification step to obtain higher throughput. These particles allow solute molecules to pass through faster and at lower pressures compared to packed beds and the capacity is higher compared to nonporous particles [8,14].

Expanded beds were developed to prevent clogging of the column [10]. An upward flow is applied to increase the space between the particles and allows coarser impurities to flow through. However, the size and density of the particles and the flow rate must be carefully balanced; if the particles are small, the flow must be limited to avoid overexpansion of the bed, while if the particles are large, the flow must be high enough to prevent sedimentation of the particles [15]. In both situations, the throughput of the bed is limited, either due to low flow rates or due to restricted diffusion reducing the adsorption capacity.

Membrane adsorbers have been developed to overcome these limitations. In these adsorbers, the adsorbent particles are fixed in a porous matrix or the matrix itself acts as the adsorbent [16]. The target substances are adsorbed on the adsorptive moieties in the membrane adsorber while the solvent with nonbinding and coarse impurities permeates through the pores [17]. Within this porous structure, convective mass transport takes place, which allows operation at higher flow rates compared to diffusion-controlled packed-bed chromatography [8,18]. This leads to a reduced pressure drop and facilitates the scale-up of the membrane adsorber technology [9]. However, most membrane adsorbers made by modifying micro/macroporous membranes have a low adsorption capacity due to a low surface area and a large pore size distribution [12]. A variance in porosity causes a preferential flow of the solute molecules through the larger pores resulting in an early breakthrough [8,12].

**Figure 1:**
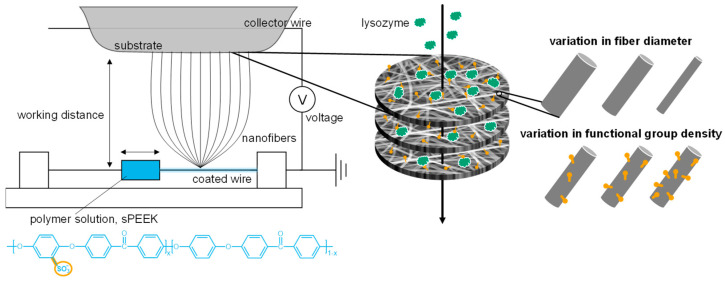
Schematic representation of the fabrication process for sPEEK membrane adsorbers using a wire-electrospinning device, along with a visualization of the factors studied for their impact on performance.

Electrospinning was introduced to enhance the surface area and versatility of membrane adsorbers, allowing the creation of nanofibrous porous mats with tailor-made functionalities to selectively recover valuable components [19]. This technique uses an electrostatic force to overcome the surface tension of a polymer solution, converting it into a fiber structure that is deposited on a collector paper forming a porous mat (Figure 1) [20,21]. These mats can be stacked with a random overlay orientation of the fibers to decrease the effective pore size distribution and achieve an even flow dispersion [22]. Electrospun membrane adsorbers offer a promising cost-effective platform technology for resource recovery. This is because electrospinning gives opportunities to (1) tailor the selectivity by functionalization of the electrospun membranes through polymer blending, functional particle embedding during electrospinning or chemical post-functionalization; (2) control permeability by adjusting the bed height, porosity, and fiber diameter such that nonbinding and coarser impurities easily elute through the bed while the desired components can bind to the (functionalized) electrospun fibers; and (3) facilitate easy production and linear scale-up [19,23,24,25]. Recent progress made in the use of electrospun nanofibers for membrane adsorbers is discussed in several review papers [9,19,23,24]. So far, the versatility of electrospun membrane adsorbers has been studied primarily in terms of design, fabrication, and type of functionalization [19]. However, many of these adsorbers require multistep synthesis for functionalization [26,27,28,29,30,31,32], which limits their entrance into industry and the market. To overcome this, alternative fabrication routes, such as the use of pre-functionalized polymers should be explored [33]. Systematic studies to tailor the performance by controlling electrospinning conditions are limited [19].

This study develops electrospun sulfonated poly(ether ether ketone) (sPEEK) membrane adsorbers that eliminate the need for any post-functionalization steps due to the inherent presence of the functional sulfonate groups in the polymer (Figure 1). The sulfonate groups allow selective binding of the model protein lysozyme under neutral pH through electrostatic interactions. The negatively charged strong acidic sulfonic acid groups and the positively charged lysozyme (with an isoelectric point of 11.35) are attracted to each other by Coulombic interactions [34,35]. Other interactions, such as hydrogen bonding, hydrophobic interactions, and van der Waals forces, also contribute to the binding affinity between the protein and the membrane adsorber [35,36,37]. The choice of lysozyme as the model protein in this study is due to its stability, antimicrobial properties, and is a natural preservative, which makes lysozyme an ideal test molecule and is therefore widely studied [38]. Most important, the versatility of electrospinning is explored with a focus on the effect of fiber thickness or functional-group density on the adsorber performance (Figure 1). The sPEEK fiber thickness was tailored by varying the concentration of the polymer solution and fine-tuning the process parameters of the electrospinner [20,39,40]. sPEEK membrane adsorbers with thicknesses of 90 ± 18 nm, 132 ± 27 nm, and 166 ± 18 nm were investigated. Additionally, the effect of the functional-group density on the binding capacity was studied by creating sPEEK-based membrane adsorbers from sPEEK with different sulfonation degrees (52%, 62%, and 72%).

## 2. Materials and Methods

### 2.1. Materials

Sulfonated poly(ether ether ketone) (sPEEK) was purchased from FumaTech-BWT GmbH, Bietigheim-Bissingen, Germany, Fumion^®^ with sulfonation degrees of 52% (sPEEK-52, x = 0.52 in Figure 1), 62% (sPEEK-62), and 72% (sPEEK-72). Dimethylacetamide (DMAc) was supplied by Sigma–Aldrich, Darmstadt, Germany and N-methylpyrrolidone (NMP) by Biosolve B.V., Valkenswaard, The Netherlands. To dry the solvents molecular sieves were used (4 Å, Sigma–Aldrich, Darmstadt, Germany). Hydrochloric acid (HCl, Supelco^®^ from Sigma–Aldrich, Darmstadt, Germany), sodium hydroxide (NaOH, VWR Chemicals, Boxmeer, The Netherlands), and sodium chloride (NaCl, Sanal^®^ P, AkzoNobel, Deventer, The Netherlands) were used for membrane pretreatment and/or characterization. Demineralized water was obtained from an Elga water purification system from Veolia, Weert, The Netherlands. Lysozyme (LZ) from hen egg white (Mw ~ 14,600, Fluka analytical), phosphate buffers saline (PBS) tablets (pH 7.4, 0.01 M PBS, total ionic strength 0.15 M, Sigma–Aldrich, Darmstadt, Germany), and syringe filter holders (25 mm, Sartorius, Goettingen, Germany) were used to measure the membrane performance.

### 2.2. Preparation of Electrospun Membranes

For electrospinning, the polymer is dissolved in a solvent. During the spinning process, the polymer solidifies, thereby forming a fiber. Both the polymer, solution, and process conditions define, e.g., the dimensions of the fiber. The driving force in electrospinning is the electrical field built between the polymer supply and the collector. This electric charge causes instability in the polymer solution because of the induction of charges on the polymer and the charge builds up mainly at the surface of the liquid, destabilizing the meniscus of the droplet on the wire. When the electric charge overcomes the surface tension, a jet is formed.

Before solution preparation, the sPEEK polymers were dried in the vacuum oven at 80 °C for six hours. The dried sPEEK-52 was used to prepare a 22 weight-% (wt %) solution using NMP as a solvent, which had been dried using molecular sieves. The solution was placed on the roller bench for at least 24 h. Additionally, the polymer solution was placed in an ultrasonic bath at 25 °C for at least 4 h to break up any gel particles that may be present in the solution. Polymer solutions of 17–25 wt % sPEEK-62 and sPEEK-72, with solubility properties distinct from sPEEK-52, were prepared using dried DMAc as a solvent and placed on the roller bench for at least 15 h. While the polymer solution was still hazy, the solution was sonicated to obtain a homogeneous transparent solution. Electrospinning was performed using a wire-electrospinning device (Nanospider NS LAB, Elmarco, Liberec, Czech Republic). The relative humidity and temperature of the electrospinning chamber were controlled (desiccant dehumidifier system, ML270PLUS, Munters, Den Haag, The Netherlands).

The polymer solutions were electrospun from a carrier with an orifice of 0.8 mm moving along the working wire electrode at a speed of 150 mm/s. The applied voltage between the working and collecting electrode (working distance was set to 150 mm) was set at 80 kV. The substrate was not moving and its distance to the collecting electrode was set at 25 mm. Nanofibers were produced at 22 ± 0.5 °C under 25 ± 1% relative humidity, except for 23.4 wt % sPEEK-72, which was produced under 20 ± 1% relative humidity. The obtained spunbound membranes were placed for conditioning in 1 M HCl on a shaking plate for one hour to ensure that all sulfonate groups have an H^+^ as their counterion. Then, the membranes were rinsed in demineralized water by refreshing the water multiple times until a neutral pH was obtained. Next, the membranes were dried in a vacuum oven at 80 °C for at least 6 h.

### 2.3. Membrane Characterization

#### 2.3.1. Scanning Electron Microscopy (SEM)

The morphology of the fabricated membranes was evaluated using SEM (JEOL IT-100, Nieuw-Vennep, The Netherlands) with 10 kV accelerating voltage and probe current setting 32. All measured samples were platinum coated for 60 s at 40 mA using a sputtercoater (JFC-2300HR, JEOL, Nieuw-Vennep, The Netherlands). Fiber dimensions were measured on at least 100 spots at 10.000× magnification using ImageJ software.

#### 2.3.2. BET Surface Area

The Brunauer–Emmett–Teller (BET) specific surface area of the electrospun membranes was determined by N_2_ physisorption at liquid N_2_ temperature (−196 °C) with a Micromeretics TriStar II (Eindhoven, The Netherlands) using the Plus 3.03 software with optimized BET calculation. Prior to the measurement, samples of ~0.1 g were outgassed for 20 h at 80 °C under vacuum.

#### 2.3.3. Capillary Liquid Porometry

The pore size distribution was studied by porometry (Porolux 500, Porometer, Nazareth, Belgium) on a sample with a diameter of 25 mm. Nitrogen gas was used as the pressurizing agent, wetting was done with Porofil^®^ (15.9 dyn/cm, supplied by Porometer, Nazareth, Belgium). From each membrane, two samples were measured with the following settings: shape factor 0.715, pressure increasing slope 120 s/bar, final pressure 6 bar, number of measurements steps wet curve 50, and number of measurements steps dry curve 25.

#### 2.3.4. Water Uptake and Swelling

The sPEEK water uptake and swelling (%) were measured on cast sPEEK films in duplicates. Hereto the SPEEK solutions that were prepared for the electrospinning were cast on a glass plate using a 500 µm casting knife for sPEEK-52 and sPEEK-62, and a 300 µm casting knife for sPEEK-72. Then the films were dried for two days in a nitrogen box and six days in a nitrogen oven at 120 °C. Then the films were immersed in water for three days to ensure a fully saturated water uptake. Subsequently, the films were carefully wiped with paper to remove excess solution and weighed. The films were put in a vacuum oven at 60 °C for 20 h. Once again, the films were weighed, and the water uptake was calculated using Equation (1) and the thickness using Equation (2).

(1) $$\mathrm{Water}\;\mathrm{uptake}=\frac{m_{\mathrm{wet}}-m_{\mathrm{dry}}}{m_{\mathrm{dry}}}\cdot 100\%$$

where $m_{\mathrm{wet}}$ is the weight of the wet membrane (g) and $m_{\mathrm{dry}}$ is the weight of the dried membrane (g).

(2) $$\mathrm{Swelling}\;\mathrm{thickness}=\frac{t_{\mathrm{wet}}-t_{\mathrm{dry}}}{t_{\mathrm{dry}}}\cdot 100\%$$

where $t_{\mathrm{wet}}$ is the thickness of the wet membrane (µm) and $t_{\mathrm{dry}}$ is the thickness of the dried membrane (µm).

#### 2.3.5. Ion-Exchange Capacity

The ion-exchange capacity reflects the number of functional cationic groups that are available for ion exchange. The ion-exchange capacity of the electrospun membranes was determined through acid–base titration as reported by Park et al. [41]. First, the membranes were immersed overnight in 1 M HCl to convert them into the H^+^ form. After, the membranes were thoroughly rinsed with demineralized water to remove the unbound H^+^ ions from the spunbound membranes. Subsequently, the membranes were soaked three times for one hour in 15 mL 2 M NaCl to exchange Na^+^ for H^+^. For each membrane sample, the combined salt solutions were titrated with 0.01 M NaOH using a titrator from Metler Toledo with sensor DGi115-SC. The ion-exchange capacity (meq/g dry membrane) was calculated using the following Equation (3):

(3) $$\mathrm{Ion-exchange}\;\mathrm{capacity}=\frac{M_{\mathrm{NaOH}}\cdot V_{\mathrm{NaOH}}}{m_{\mathrm{dry}}}$$

where $M_{\mathrm{NaOH}}$ is the molar concentration of the sodium hydroxide solution (M), $V_{\mathrm{NaOH}}$ the volume of sodium hydroxide needed to titrate the acid (mL) and $m_{\mathrm{dry}}$ the dry mass of the membrane (g). All ion-exchange capacity measurements were executed in triplicate.

### 2.4. Membrane Performance

#### 2.4.1. Static Lysozyme Adsorption

The maximum adsorption capacity of lysozyme on the membrane adsorbers was evaluated through static adsorption experiments. First, lysozyme solutions with concentrations ranging from 0–2.5 mg/mL in PBS buffer were made and measured by UV-vis spectroscopy at 280 nm (Shimadzu UV-1280, ‘s-Hertogenbosch, The Netherlands). The sPEEK membranes (~0.02 g) were immersed in 2 mL lysozyme solution with the predetermined concentrations (in duplicates). The adsorption experiments were carried out at room temperature for 20 h on a shaking plate to ensure equilibrium and the concentrations were measured again. The amount of adsorbed lysozyme (mg/g) was calculated using the following Equation (4):

(4) $$\mathrm{Adsorbed}\;\mathrm{lysozyme}=\frac{\left(C_{0}-C_{t}\right)\cdot V}{m}$$

where $C_{0}$ is the initial concentration and $C_{t}$ the equilibrium concentration of lysozyme in solution (mg/mL), V is the volume of the solution (mL), and m the mass of the membrane (g). The adsorption isotherm follows the Langmuir isotherm, which is described by the following Equation (5):

(5) $$q_{e}=\frac{Q_{m}\cdot K_{d}\cdot C_{e}}{(1+K_{d}\cdot C_{e})}$$

where $q_{e}$ is the equilibrium adsorption capacity (mg/g), $C_{e}$ is the equilibrium concentration (mg/mL), and $K_{d}$ is the equilibrium constant (mL/mg). $Q_{m}$ is the maximum adsorption capacity using a curve fitting (mg/g).

#### 2.4.2. Dynamic Lysozyme Adsorption

For the determination of the dynamic adsorption capacity, 5–20 membrane discs with a diameter of 25 mm each were cut and stacked in a filter holder (total mass membrane 0.05–0.15 g). The membrane mass available for adsorption was determined as the mass enclosed within the o-ring. The filter holder was connected to a syringe filled with PBS solution to flush the system and eliminate any potential contaminants. The flow velocity was controlled with a syringe pump (Chemyx Inc. Fusion 200, Stafford, TX, USA) and set at 1.0 mL/min for the adsorption step. The syringe was filled with 0.5 mg/mL lysozyme in PBS solution and the permeate was collected in fractions of ~0.7 mL. The concentration of lysozyme was determined by UV-vis spectroscopy at 280 nm. When the concentration in the permeate exceeded 10% of the feed concentration (breakthrough point) the pump was stopped. The adsorption capacity was determined by interpolation of the adsorption curve at 10% breakthrough. The dynamic adsorption was executed for most samples in duplicates, and the adsorption capacity showed an error margin of ≤11%. For the washing step, the lysozyme in the syringe was replaced by PBS buffer solution to remove the unbound lysozyme. The flow velocity in this washing step was set at 0.5 mL/min for practical reasons. For desorption, the syringe was filled with 0.5 M NaCl in PBS solution and the flow velocity was set at 1 mL/min. The amount of desorbed lysozyme in the desorption buffer was determined by UV-vis spectroscopy at 280 nm and the recovery is calculated using the following Equation (6):

(6) $$\mathrm{Recovery}=\frac{P_{D}}{P_{L}-P_{\mathrm{AW}}}\cdot 100\%$$

where $P_{D}$ is the amount of protein removed from the membrane stack in the desorption step (mg), $P_{L}$ is the amount of protein loaded on the membrane stack (mg), and $P_{\mathrm{AW}}$ is the amount of unbound protein eluted in the adsorption and washing step (mg). 

## 3. Results and Discussion

### 3.1. Electrospun sPEEK Membrane Adsorbers

**Figure 2:**
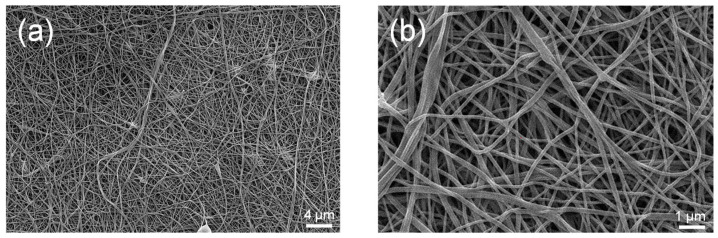
SEM images of sPEEK-62 membrane adsorbers in the H^+^ form made by wire-electrospinning using a 19 wt % polymer solution; (**a**) 2500× magnification and (**b**) 10,000× magnification.

Membrane adsorbers with sulfonic acid functional groups were fabricated by wire-electrospinning a 19 wt % sPEEK-62 in DMAc solution. SEM images of the nanofibrous mats after the conditioning step show a uniform fiber morphology with an average fiber diameter of 132 ± 27 nm (Figure 2) with an associated BET surface area of 12.3 ± 2.1 m^2^/g. The obtained fiber diameter is relatively small compared to the data provided in the review paper by Yang et al. (ranging from 150–15,000 nm) [9]. As a result, the surface area is relatively high compared to values reported in literature (4–7 m^2^/g) [9].

**Figure 3:**
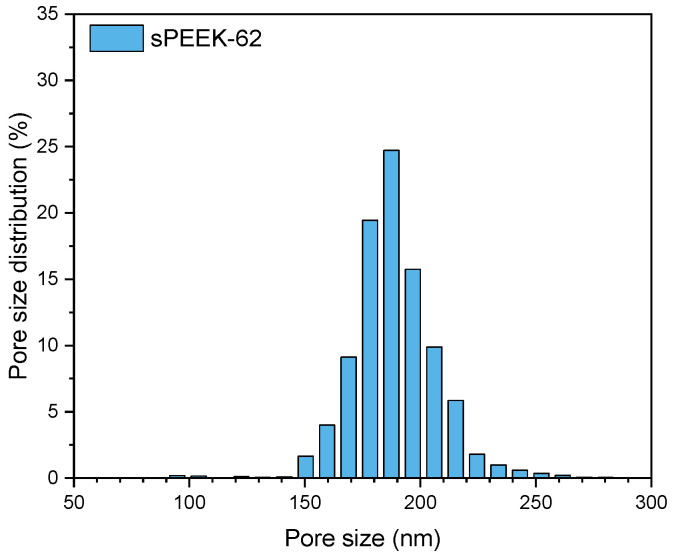
Pore size distribution measured with capillary liquid porometry of sPEEK-62 membrane adsorbers in the H^+^ form made by wire-electrospinning using a 19 wt % polymer solution.

Achieving a uniform plug-flow velocity through the membrane adsorber and utilizing its complete adsorption capacity relies on a narrow pore size distribution. The pore size distribution of this sPEEK-62 membrane is shown in Figure 3. The pores of the membrane are almost 50 times larger than the size of a lysozyme molecule (4.5 × 3 × 3 nm), allowing convective transport of the lysozyme without clogging the pores [42].

**Figure 4:**
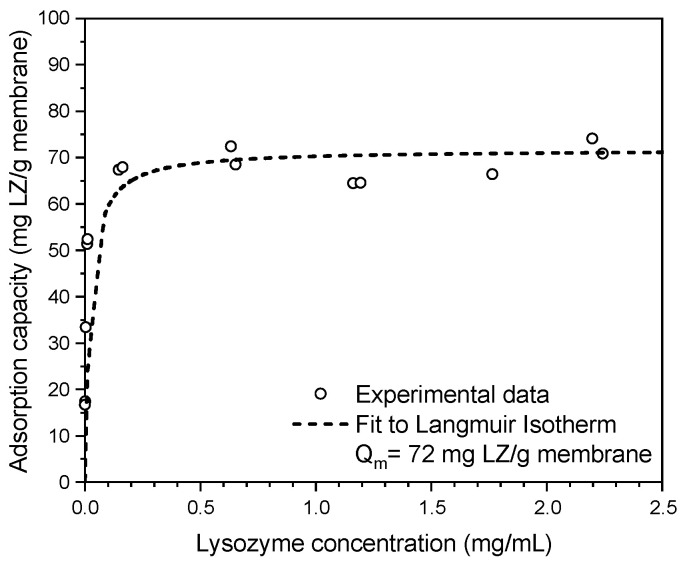
Static adsorption isotherm (at equilibrium concentration, C_e_) of lysozyme (LZ) in PBS buffer (pH = 7.4) on an electrospun sPEEK-62 membrane adsorber with a maximum adsorption capacity (Q_m_) of 72 mg LZ/g membrane.

The membrane adsorber performance is studied by measuring the static adsorption capacity using lysozyme as a model protein (Equation (4), Figure 4). Lysozyme is positively charged at neutral pH enabling electrostatic binding with the negatively charged sulfonic acid groups of sPEEK [34]. The experimental results in Figure 4 have been fitted using the Langmuir adsorption isotherm expression from Equation (5), which gives a maximum equilibrium adsorption capacity Q_m_ of 72 mg/g.

**Figure 5:**
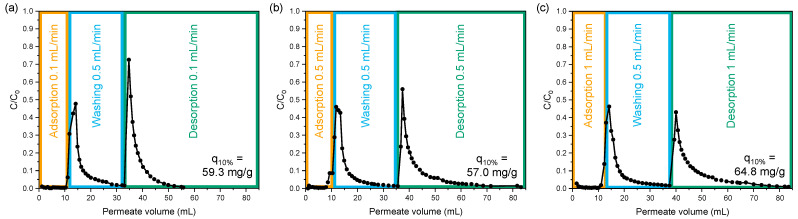
Dynamic lysozyme adsorption and desorption curves of electrospun sPEEK-62 membrane adsorbers with flow velocities of (**a**) 0.1 mL/min, (**b**) 0.5 mL/min, and (**c**) 1 mL/min (0.5 mg LZ/mL, pH 7.4); q_10%_ is the adsorption capacity at 10% breakthrough.

The dynamic adsorption capacity of the membrane adsorber is studied by measuring the breakthrough curve, showing the lysozyme load (permeate volume in the adsorption step) versus the eluent concentration (Figure 5). Initially, no lysozyme is detected in the eluent, indicating full adsorption on the membrane adsorber. When the adsorber becomes saturated, with the majority of the adsorption sites occupied, the first lysozyme molecules start to break through, causing a lysozyme increase in the eluent. By convention, the dynamic adsorption capacity is determined at 10% breakthrough (q_10%_) to minimize product loss. Dynamic adsorption experiments of a stack of 20 membranes loaded with 0.5 mg/mL lysozyme at a flow of 0.1 mL/min show an adsorption capacity at 10% breakthrough of 59.3 mg/g (Figure 5a). This corresponds to a lysozyme adsorption capacity that is comparable to the static adsorption capacity at C_e_ of 0.05 mg/mL, being the concentration at 10% breakthrough. This means full utilization of the lysozyme adsorption capacity and indicates an almost ideal plug flow in the membrane stack. Furthermore, the dynamic adsorption capacity at varying flow velocity (from 0.1 mL/min to 1 mL/min) showed only small deviations (within the expected-error margin, as reported in the experimental section) and was independent of the set flow velocities, confirming that convective mass transport is dominant in the membrane adsorber stack (Figure 5a–c).

**Figure 6:**
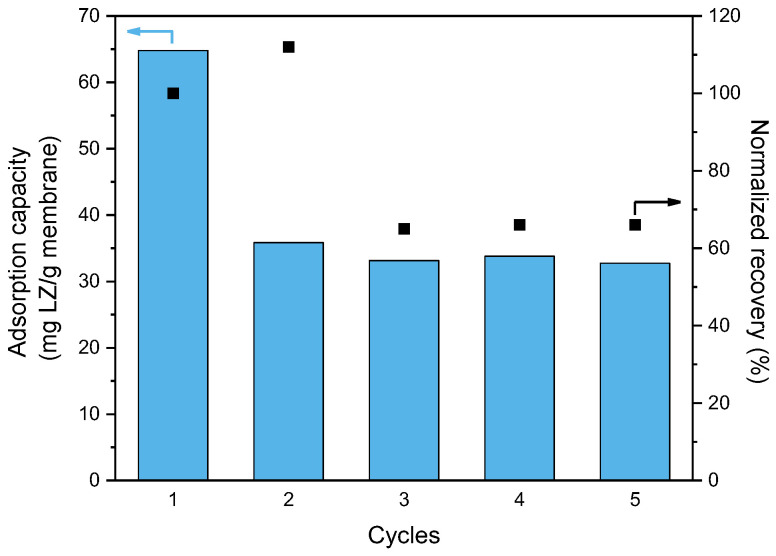
Dynamic lysozyme adsorption capacity and recovery of sPEEK-62 membrane adsorber for 5 cycles (flow velocity 1.0 mL/min, 0.5 mg LZ/mL, pH 7.4). The recovery is normalized with the recovery of the first cycle.

The peak in the desorption step shows the amount of lysozyme removed from the membrane adsorber using 0.5 M NaCl in PBS buffer, which is 31 ± 2% of the total adsorbed lysozyme, regardless of the flow velocity. This suggests that lysozyme adsorbs on the membrane by two modes of adsorption, namely reversible electrostatic interactions, and irreversible hydrophobic interactions. Adsorption with these two modes is supported by the study of Dismer et al. who showed that in the case of a resin with a hydrophobic backbone (polystyrene) functionalized with sulfonic acid groups (such as our sulfonated PEEK), lysozyme binding occurs through electrostatic and hydrophobic interactions [43,44]. Furthermore, computer simulations by Yu et al. indicate that when the lysozyme binds with its hydrophobic region with only four positive residues in its surroundings (in contrast to the other hydrophobic region surrounded by 13 positive residues), desorption is inhibited [45]. Once the hydrophobic binding sites are covered with lysozyme only the reversible electrostatic binding sites remain available, which leads to an overall decrease in lysozyme adsorption capacity in the second and subsequent cycles (Figure 6). The recovery, normalized with the recovery of the first cycle, shows a small increase in the second cycle. This could be attributed to a binding rearrangement from hydrophobic to electrostatic binding, likely due to the change in ionic strength between the desorption step of cycle 1 (0.65 M) and the adsorption step of cycle 2 (0.15 M). At low ionic strength, electrostatic interactions are dominant in protein adsorption, while at higher ionic strength, the charges are screened by the ions in solution, and hydrophobic interactions become dominant in protein adsorption [46,47]. From the third cycle onward, the adsorption capacity and recovery remained fairly constant, indicating that the hydrophobic binding sites are occupied and interactions in these cycles primarily occur at the reversible electrostatic binding sites, i.e., sulfonic acid groups. These findings demonstrate that sPEEK-62 membrane adsorbers are reusable and capable of operating at a constant efficiency after the third cycle.

### 3.2. The Effect of Fiber Diameter

**Figure 7:**
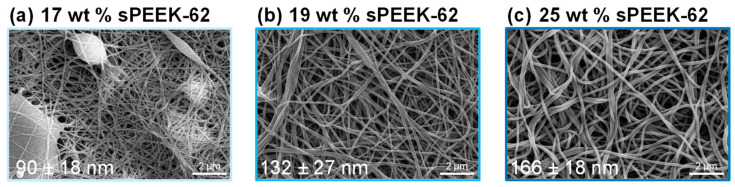
SEM images of sPEEK-62 membrane adsorbers in the H^+^ form made by wire-electrospinning using polymer concentrations of (**a**) 17 wt %, (**b**) 19 wt %, and (**c**) 25 wt %. Average fiber diameters are given in the bottom left corner of each image.

The versatility of electrospinning is explored with a focus on the relationship between fiber diameter and performance. The sPEEK-62 membrane adsorbers with different fiber diameters were fabricated by varying the concentration of the polymer solution in the wire-electrospinning process. The SEM images of the fabricated sPEEK membranes after proton exchange show a variation in fiber diameter from 90 ± 18 nm to 166 ± 18 nm with increasing polymer concentration (Figure 7). Based on our experience, this is the widest range of fiber diameters achievable through wire-electrospinning with this material because lower concentrations of polymer solution yield fibers with beads (as already observed in SEM with 17 wt % sPEEK-62), and higher concentrations are too viscous and prone to gelation, making them unsuitable for electrospinning [25,40]. Small optimizations in the electrospinning process parameters could be made to enlarge the variations in fiber diameter, although it is known that the polymer concentration has the largest effect on fiber diameter [39]. Despite the considerable variations in fiber diameter observed with SEM, only minor variations in the surface area were measured with BET. Specifically, the measured surface areas were 11.2 ± 0.7 m^2^/g, 12.3 ± 2.1 m^2^/g, and 16.5 ± 1.3 m^2^/g for the sPEEK-62 membrane adsorbers created with 17 wt %, 19 wt %, and 25 wt % polymer solution, respectively. Surprisingly, the membrane adsorber with the largest fiber diameter has the highest surface area, which could be due to the submicron-scale surface roughness of the fibers. This surface roughness is likely a result of buckling instability during the electrospinning process, where the skin layer formed on the polymer fibers collapses as the solvent evaporates, leading to a wrinkled surface. This phenomenon occurs more frequently with thicker fibers, which have a reduced surface-to-volume ratio and therefore longer drying times, increasing the likelihood of skin-layer formation [48,49,50]. Consequently, it is more probable that the thickest fiber has the greatest surface roughness, even though it cannot be observed with SEM. In contrast, the membrane adsorber with the smallest fiber diameter could have a reduced surface area due to the formation of beads.

**Figure 8:**
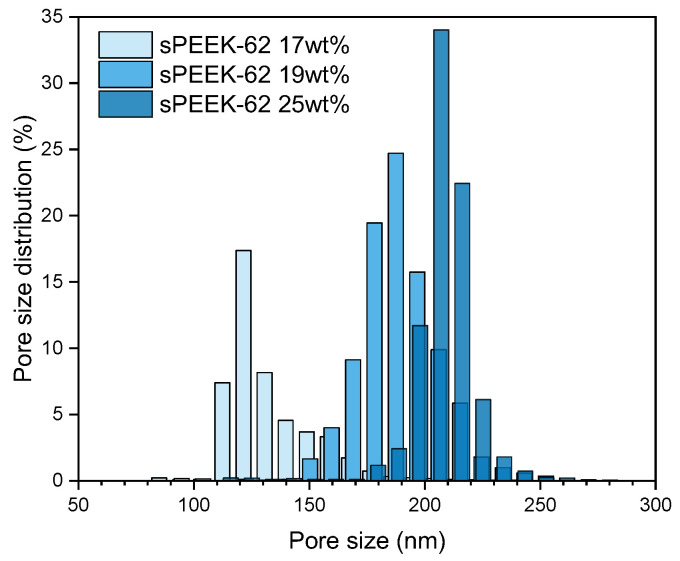
Pore size distribution of sPEEK-62 membrane adsorbers with different fiber diameters (made with polymer concentrations of 17 wt %, 19 wt %, and 25 wt %) as measured with porometry.

For a fair comparison of the dynamic adsorption capacity, it is important to take the pore size distribution into account as it influences the flow distribution through the membrane adsorbers. The porometry results show that the pore size distribution is in the same range for all three samples (Figure 8) and will probably be narrowed down when using a stack of membranes [26,28]. It is worth noting that larger fiber diameters correspond to larger pore sizes. This is because thicker fibers are created by pulling more material from the wire, which makes the space between the fibers wider resulting in larger pores [51,52,53,54].

Dynamic adsorption measurements with lysozyme are performed to evaluate the performance of all three sPEEK-62 membrane adsorbers. The adsorption capacity results (in mg/g) show that there is no correlation between adsorption capacity and fiber diameter since the total surface area of the membrane did not change with a change in the fiber diameter (Table 1). When the adsorption capacity is normalized for the surface area, a slight decrease is observed for the sPEEK-62 membranes produced with higher polymer concentrations. As the material properties remain constant for these membranes and the average pore size is larger for those produced with higher polymer concentrations, this suggests that the amount of lysozyme adsorbed per surface area is slightly decreased due to the larger pores. This could be attributed to the longer time required for the lysozyme to reach the surface of the adsorber. Despite this, the recovery values for all three membrane adsorbers were similar, as the material properties, i.e., ratio electrostatic and hydrophobic interactions, are identical for all three sPEEK-62 membranes.

### 3.3. The Effect of Sulfonation Degree

The versatility of sPEEK membrane adsorbers is explored by using the sPEEK of variable sulfonation degrees (52%, 62%, and 72%), as validated by H-NMR using the method of Zaidi et al. [55]. The sulfonation degree determines the number of functional groups present for adsorption. First, the properties of the different sPEEK polymers are studied by measuring the water uptake and swelling behavior of cast sPEEK films (Table 2). The water uptake and swelling thickness properties of sPEEK-52 and sPEEK-62 are rather similar, whereas sPEEK-72 shows a substantial increase in both the water uptake and the swelling thickness. This is supported by Zaidi et al. where they show that the water uptake increases linearly with a sulfonation degree up to 65% (with a reported water uptake of 33%), followed by a rapid increase above a sulfonation degree of 70% (with a water uptake of 47% at a sulfonation degree of 72%) [55]. The high density of SO_3_H groups in the highly sulfonated sPEEK can form clusters that absorb more water, explaining the rapid increase in water uptake [55,56]. The high water uptake results in severe swelling and gelation affecting the dimensional stability; at higher sulfonation degree values (100%) the polymer becomes even water soluble. Therefore sPEEK-72 was selected as the upper limit in sulfonation degree.

The experimentally determined ion-exchange capacity of the electrospun membranes is reported in Table 2. The quantity of accessible functional groups increases using sPEEK with a higher sulfonation degree. It should be noted that the ion-exchange capacity represents the overall number of functional cationic groups available for small ions that can penetrate inside the fiber. However, it is unlikely that lysozyme, with a dimension of approximately 4.5 × 3 × 3 nm [42], has access to the subsurface sulfonic acid groups, resulting in a lower quantity of functional groups available for lysozyme binding compared to the measured ion-exchange capacity.

**Figure 9:**
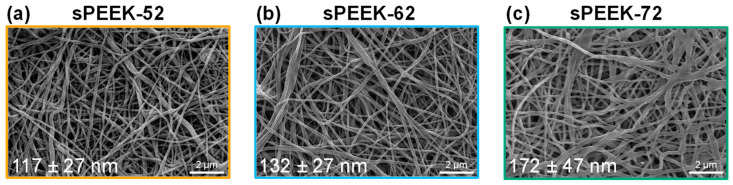
SEM images of membrane adsorbers in the H^+^ form made by wire-electrospinning using sPEEK with different sulfonation degrees of (**a**) 52%, (**b**) 62%, and (**c**) 72%. Average fiber diameters are given in the bottom left corner of each image.

The properties of the sPEEK polymer change with the sulfonation degree. In the electrospinning process, the charge mainly accumulates at the surface of the liquid, which destabilizes the meniscus of the droplet and changes the jet formation. For this reason, the electrospinning parameters had to be adjusted for every polymer solution (22 wt % sPEEK-52, 19 wt % sPEEK-62, and 23 wt % sPEEK-72), as described in the experimental section. Despite efforts to achieve uniform thickness in the sPEEK fibers of varying sulfonation degrees, variations in fiber diameter were observed in the SEM images (Figure 9). Additionally, the fusion of some fibers was observed in the sPEEK-72 membrane, which may be attributed to the high degree of swelling of this polymer. As a result, an increased average diameter is measured compared to the pristine membrane, which had an average diameter of 158 ± 35 nm (SEM image not shown).

The surface areas of the membranes, as measured with BET, were 14.9 ± 2.9 m^2^/g, 12.3 ± 2.1 m^2^/g, and 9.5 ± 2.4 m^2^/g for sPEEK-52, sPEEK-62, and sPEEK-72, respectively. The BET measurements show a reduction in surface area for the sPEEK-72 membrane compared to the sPEEK-62 membrane, likely due to the fusion of fibers, as observed in SEM. Additionally, the sPEEK-52 membrane shows an even higher surface area than the sPEEK-62 membrane with the smallest fiber diameter (11.2 ± 0.7 m^2^/g, 17 wt % sPEEK-62, discussed in the previous section), despite having a slightly higher fiber diameter. This can be attributed to a more homogenous fiber formation with sPEEK-52 resulting in a higher surface area.

**Figure 10:**
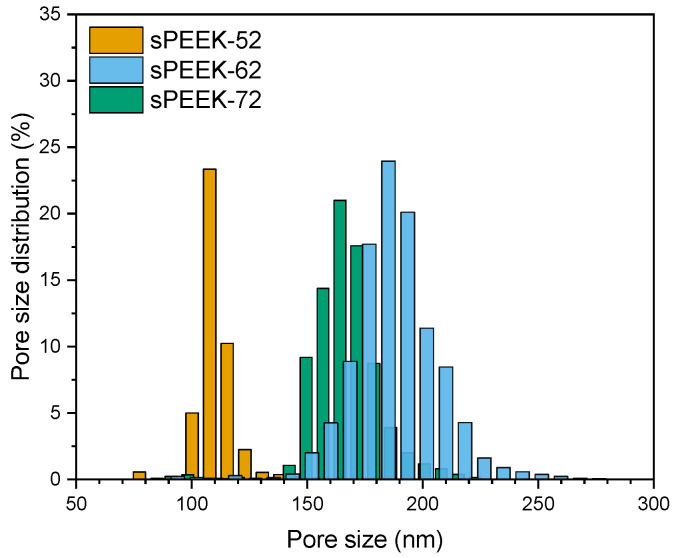
Pore size distribution of the sPEEK membrane adsorbers in the H^+^ form with different sulfonation degrees (52%, 62%, and 72%) as measured with porometry.

Additionally, porometry showed that the sPEEK-52 membranes had a narrower pore size distribution, indicating more homogeneity of the fibrous structure (Figure 10). The average pore size of sPEEK-72 is slightly smaller than that of sPEEK-62, although sPEEK-72 has a higher fiber diameter. This reduction in pore size is likely due to the higher swelling of sPEEK-72.

The effect of sulfonation degree on the membrane performance is studied by measuring the dynamic lysozyme adsorption and desorption curves. The results show that the sulfonation degree, i.e., the number of functional groups per unit mass, did not directly influence the lysozyme adsorption capacity in mg/g, as sPEEK-52 with the lowest functional-group density exhibits the highest adsorption capacity (Table 3). However, when considering the adsorption capacity in mg/m^2^, which normalizes for surface area, the adsorption capacity increased with increasing sulfonation degree.

The literature has shown that lysozyme with a net charge of +8 at neutral pH (17 positively charged residues and 9 negatively charged residues) binds with four positive key residues on the negatively charged surface of the membrane adsorber [44,46]. The orientation of lysozyme on the membrane surface depends on the ligand type, ionic strength, and surface interactions, and can be side-on (with its long axis parallel to the surface) or, more efficiently, end-on [44,46]. Depending on the orientation, the lysozyme monolayer surface coverage varies from 2–3 mg/m^2^ [57]. The adsorption capacity in mg/m^2^ shows that all our membranes are completely covered by lysozyme, without being limited in the number of functional groups. In fact, the coverage seems to exceed that of a monolayer. This is due to the discrepancy in measurement conditions. The BET is measured in the dry state, while the adsorption is measured in the wet state. The swelling of the fibers creates more spaces for lysozyme adsorption and increases the surface area beyond what is measured in the dry state using BET. This also explains the high adsorption capacity in mg/m^2^ of the sPEEK-72 membrane adsorber, as severe swelling of this sample is observed increasing the surface area in the wet state. It is worth noting that the standard deviation for this sample is high, which may be attributed to the swelling resulting in fiber fusion which can vary between samples. Overall, the adsorption data, combined with the surface area, suggest that the swelling, which is linked to the sulfonation degree, is the main factor that increases the adsorption capacity.

However, this positive effect of swelling is not unlimited; with an increase in sulfonation degree, fiber swelling can increase the flow resistance. Pump stalling has been observed when the flow rates exceeded 1 mL/min, which limits the throughput and reduces the efficiency of the adsorption process. Moreover, excessive fiber swelling can create dead zones and promote channeling that induces a decrease in the dynamic adsorption capacity.

The lysozyme recovery is comparable for the sPEEK-52 and sPEEK-62 membranes, but it increases for the sPEEK-72 membrane. This can be attributed to the higher number of charged groups in the sPEEK-72 membrane, which also makes the surface more hydrophilic. As a result, the reversible electrostatic interactions are favored over the irreversible hydrophobic interactions, leading to easier desorption and a higher lysozyme recovery.

To put this work into perspective, the adsorption capacity of our membrane is similar to the adsorption capacity of Sartobind^®^ S (52 mg/g), a commercial membrane adsorber with grafted polymers containing sulfonic acid groups [58]. However, in the literature, membrane adsorbers that surpass the adsorption capacity of our sPEEK are reported [59,60]. The drawback of these membrane adsorbers is that they require functionalization through multistep synthesis, whereas our sPEEK membranes can be directly used after electrospinning, making them easy to scale-up. Electrospinning offers the opportunity to tailor the fiber diameter; however, only small changes in surface area and, therefore, adsorption capacity are observed. To enhance the adsorption capacity, co-electrospinning of functionalized adsorptive/affinity particles can be explored, which offer additional selective binding sites [17,23,24,61]. Another approach is grafting polymer brushes, which enable multilayer stacking of proteins, leading to high effective surface areas [62,63,64,65]. Increasing the number of functional groups is also a strategy for enhancing adsorption capacity, as done in this study by increasing the number of functional groups. However, an increased degree of sulfonation did not result in an increased lysozyme adsorption capacity. This is because lysozyme covers many more charged groups than itself needs to bind on the membrane adsorber; therefore, in this specific case of lysozyme binding, there is no advantage in increasing the charge density of the membrane adsorber. Conversely, smaller target molecules that do not cover the excess charge and that do not experience steric hindrance can benefit from a higher sulfonation degree. From this point of view, testing sPEEK membrane adsorbers has provided valuable insights into the relationship between surface area, swelling, and adsorption capacity.

## 4. Conclusions

In this work, a wire-electrospun sPEEK membrane adsorber was developed without the need for any additional functionalization step. This sPEEK-based membrane adsorber has a dynamic lysozyme adsorption capacity (at 10% breakthrough) of 59.3 mg/g. This is comparable to the static adsorption capacity at the same concentration, demonstrating full utilization of the lysozyme adsorption capacity and indicating an almost ideal plug flow in the membrane stack. The dynamic adsorption capacity is independent of the flow rate (varying from 0.1 to 1 mL/min), indicating that convective mass transport is dominant, and adsorption is not limited by diffusion. Cycling the dynamic adsorption capacity experiment suggests that lysozyme binds by two modes of adsorption, namely reversible electrostatic interactions and irreversible hydrophobic interactions. Furthermore, this experiment demonstrates that the sPEEK membrane adsorbers are reusable and capable of operating at a constant efficiency after the third cycle. The versatility of electrospinning was explored by creating nanofibrous mats with variable diameters, ranging from 90 ± 18 to 166 ± 18 nm. The dynamic adsorption capacity and surface area in this range of fiber diameters were minimally affected by variations in fiber diameter. Therefore, electrospinning offers membrane adsorbers with consistent performance, even when the process yields variations in fiber diameter. Additionally, the effect of the functional-group density on the binding capacity was studied by creating membrane adsorbers from sPEEK with different sulfonation degrees (52%, 62%, and 72%). In all presented cases, at least a monolayer coverage was obtained. This suggests that there is already an abundant number of functional groups available within the area occupied by a lysozyme molecule, and, therefore, increasing the functional-group density does not enhance the adsorption capacity. In brief, our study has demonstrated the successful development of a membrane absorber that can be immediately used for binding positively charged molecules. However, the potential for performance tuning through adjustments to fiber diameter or functional-group density is limited.

## Figures and Tables

**Table 1:** Dynamic lysozyme adsorption capacity at 10% breakthrough and recovery of sPEEK-62 membrane adsorbers with different fiber diameters (made with different polymer concentrations of 17 wt %, 19 wt %, and 25 wt %).

	17 wt % sPEEK-62	19 wt % sPEEK-62	25 wt % sPEEK-62
**BET surface area (m^2^)**	11.2 ± 0.7	12.3 ± 2.1	16.5 ± 1.3
**Adsorption capacity (mg/g)**	66.0 ± 6.4	60.1 ± 6.6	76.0 ± 3.0
**Adsorption capacity (mg/m^2^)**	5.9 ± 0.7	4.9 ± 1.0	4.6 ± 0.4
**Recovery (%)**	37 ± 11%	35 ± 6%	36 ± 3%

**Table 2:** Water uptake and swelling thickness of cast sPEEK films and the ion-exchange capacity of electrospun sPEEK membranes.

	sPEEK-52	sPEEK-62	sPEEK-72
**Water uptake (%)**	34 ± 5%	39 ± 2%	51 ± 7%
**Swelling thickness (%)**	11 ± 1%	12 ± 9%	18 ± 8%
**Ion-exchange capacity (meq/g)**	1.30 ± 0.02	1.56 ± 0.05	1.89 ± 0.09

**Table 3:** Dynamic lysozyme adsorption capacity at 10% breakthrough and recovery of sPEEK membrane adsorbers with different sulfonation degrees (52%, 62%, and 72%).

	sPEEK-52	sPEEK-62	sPEEK-72
**BET surface area (m^2^)**	14.9 ± 2.9	12.3 ± 2.1	9.5 ± 2.4
**Adsorption capacity (mg/g)**	65.7 ± 5.4	60.1 ± 6.6	63.9 ± 3.4
**Adsorption capacity (mg/m^2^)**	4.4 ± 0.9	4.9 ± 1.0	6.7 ± 1.7
**Recovery (%)**	35 ± 5%	35 ± 6%	43 ± 7%

## Data Availability

Data are contained within the article.
